# Renin gene rs1464816 polymorphism contributes to chronic kidney disease progression in ADPKD

**DOI:** 10.1186/s12929-015-0217-0

**Published:** 2016-01-11

**Authors:** Gnanasambandan Ramanathan, Ramprasad Elumalai, Soundararajan Periyasamy, Bhaskar V. K. S. Lakkakula

**Affiliations:** Department of Biomedical Sciences, Sri Ramachandra University, Chennai, India; Department of Nephrology, Sri Ramachandra University, Chennai, India; Genetic Lab, Department of Biochemistry, Sickle Cell Institute Chhattisgarh, Pt. JNM Medical College, Raipur, 492001 CG India

**Keywords:** *REN* gene, ADPKD, CKD, tag-SNPs

## Abstract

**Background:**

Autosomal dominant polycystic kidney disease (ADPKD) is a monogenic disorder and is a common genetic cause of chronic renal failure in children and adults. The enzyme renin plays a key role in the RAAS cascade and an important role in the development of hypertension and progression of renal disease in ADPKD. The present study is aimed to investigate the potential modifier effect of *REN* gene polymorphisms on the progression of chronic kidney disease (CKD) in ADPKD.

**Methods:**

We analyzed 102 ADPKD patients and 106 healthy controls from the same geographic area. FRET-based KASPar single-nucleotide polymorphism (SNP) genotyping assays for *REN* gene tag-SNPs (rs2887284, rs2368564, rs1464816, rs7521667, rs10900555, rs6693954, rs6676670 and rs11571078) were performed. Cochran*-*Armitage trend test was used to assess the potential associations between these polymorphisms and CKD stages. Haplotype frequencies and LD measures were estimated by using the software Haploview. Mantel-Haenszel stratified analysis was used to explore confounding and interaction effects of these polymorphisms.

**Results:**

Of the eight tag-SNPs genotyped, the rs10900555 polymorphism deviated from the Hardy-Weinberg equilibrium in controls. The presence of ADPKD in general was not significantly associated with the *REN* tag-SNPs included in this study. Linkage disequilibrium analysis yielded three haplotype blocks and the haplotypes of the respective blocks are not statistically different between ADPKD and controls. In multivariate analysis, the rs1464816 TG genotype showed a significant association with the advancement of CKD in ADPKD (OR = 4.80; 95 % CI = 1.30–17.82; *p* = 0.019).

**Conclusions:**

The present study provides evidence that the rs1464816 polymorphism in *REN* is associated with CKD progression in ADPKD.

**Electronic supplementary material:**

The online version of this article (doi:10.1186/s12929-015-0217-0) contains supplementary material, which is available to authorized users.

## Background

Autosomal dominant polycystic kidney disease (ADPKD) is a monogenic disorder and a common genetic cause of chronic renal failure in children and adults. It is characterized by the accumulation of fluid-filled cysts in both kidneys and other organs [1]. According to epidemiological data, ADPKD affects at least 10 million individuals worldwide. Around 10 % of the patients may develop end-stage renal disease (ESRD) during the fourth and fifth decades of their life and need renal replacement therapy by haemodialysis or transplantation. ADPKD is genetically heterogeneous: mutations in PKD1 account for 85–90 % of cases and mutations in PKD2 and undefined PKD3 account for 10–15 % of cases [2]. A striking feature of ADPKD is its intrafamilial and interfamilial phenotypic variability. The age of onset of renal disease progression in ADPKD has been observed 15 years earlier in patients from PKD1-linked families than patients from PKD2-linked families [3]. Further, considerable renal disease variability has been observed among individuals with the same PKD2 mutations. This variability supports the notion that there are additional genetic, environmental and stochastic factors that contribute to renal disease progression in ADPKD [4]. The predictive links between severities of divergent phenotypes in ADPKD have not been identified so far [5]. In about 60 % of ADPKD patients, hypertension can be noticed before identifying any decrease in the glomerular filtration rate and relates to progressive kidney enlargement in ADPKD [6]. In both male and female ADPKD patients, a significant reduction in renal disease progression was associated with significantly lower mean arterial pressure and increased use of angiotensin-converting enzyme inhibitors (ACEIs) [7]. The activation of the intrarenal renin*–*angiotensin*–*aldosterone system (RAAS) plays a major role in the pathogenesis of hypertension in ADPKD [8]. Thus, the genes involved in the RAAS have an important role in the development of hypertension and progression of renal disease. The enzyme renin plays a key role in the RAAS cascade by cleaving the precursor angiotensinogen to release angiotensin II from angiotensin I. Plasma renin activity was found to be increased in hypertensive ADPKD patients compared to control subjects [9–11]. The gene coding for renin (*REN*) is located on chromosome 1q32, spans 12.5 kb in length and encodes the 406 amino acid precursor of renin that includes a pre- and a pro-segment carrying 20 and 46 amino acids, respectively. Mature renin contains 340 amino acids and has a mass of 37 kDa [12]. Studies concerned with *REN* polymorphisms and essential hypertension revealed inconsistent results [13, 14]. The present study is aimed at unraveling the potential modifier effect of the *REN* gene tag-SNP on the progression of chronic kidney disease (CKD) in ADPKD.

## Methods

### Subjects

A total of 102 south Indian patients with ADPKD, 55.88 % of whom are men, were recruited from the Department of Nephrology of Sri Ramachandra University, Chennai, between February 2000 and June 2014. The diagnosis of ADPKD was done based on previously described Ravine ultrasound criteria [15]. From serum creatinine levels of each patient, estimated glomerular filtration rate (eGFR) was calculated using the Modification of Diet in Renal Disease (MDRD) study formula. Further among the ADPKD patients, chronic kidney disease was defined according to the Kidney Disease Outcomes Quality Initiative (KDOQI) criteria for stages of CKD and patients were divided into different stages - early stages (CKD stages 1–3) and advanced stages (CKD stages 4 and 5) by using *e*GFR [16]*.* A total 106 healthy unrelated individuals without any kidney related disease (60.38 % of whom are men) from the same geographic location were included as controls. The study was approved by the Institutional Ethical Committee of Sri Ramachandra University, Chennai, India. After obtaining written informed consent, three mL peripheral blood sample was obtained from all subjects. Genomic DNA was isolated according to the standard procedure [17].

### Genotyping

*REN* tag-SNPs (rs2887284, rs2368564, rs1464816, rs7521667, rs10900555, rs6693954, rs6676670 and rs11571078) ascertained from genotyped SNPs in a Gujarati Indians in Houston population (GIH) in phase II of the HapMap Project with a minor allele frequency (MAF) ≥0.05 and linkage disequilibrium patterns with r^2^ ≥ 0.8 were used as a cutoff (www.hapmap.org). The *KASPar* SNP Genotyping Method (KBioscience, Herts., UK) that uses Fluorescent Resonance Energy Transfer (FRET) was adopted for genotyping [18]. For developing two allele specific forward primers and one common reverse primer, 50 bp upstream and 50 bp downstream flanking sequences around the SNP were used (Additional file 1: Table S1). KASPar assays were carried out in 5 μL reactions containing 10–20 ng of genomic DNA, 0.07 μL of assay mix, 2.5 μL of 1x KASP reaction mix and 0.43 μL of distilled water. The PCR reaction was performed as follows: 15 min at 94 °C; 10 touchdown cycles of 20s at 94 °C and 60s at 65 to 57° C; and 26–35 cycles of 20s at 94 °C and 60s at 57 °C. Fluorescence detection of the reaction was performed on an ABI7900HT and the scatter plot of the allele call data was viewed using SNPViewer (http://www.lgcgenomics.com).

### Statistical analysis

The Hardy-Weinberg equilibrium was tested for each of the SNPs based on the genotyping of ADPKD patients and healthy controls. Genotypic associations of SNPs between ADPKD and controls were tested using the Cochran*-*Armitage trend test*.* Pairwise linkage disequilibrium (LD) measures (D’ and r^2^) and haplotype blocks were assessed under the default settings of the Haploview software [19]. Among the ADPKD patients, the Cochran*-*Armitage trend test was used to assess the potential associations between these polymorphisms and CKD stages. Further, multivariate logistic regression analysis was performed to adjust for the multiple risk factors*.* The Mantel-Haenszel *χ*^2^ test was performed to evaluate the influence of different genotypes on the relationship between different CKD stages and hypertension*.* All statistical analyses were performed using SPSS (version 16.0 for Windows, SPSS Inc, Chicago, IL).

## Results

**Fig. 1:**
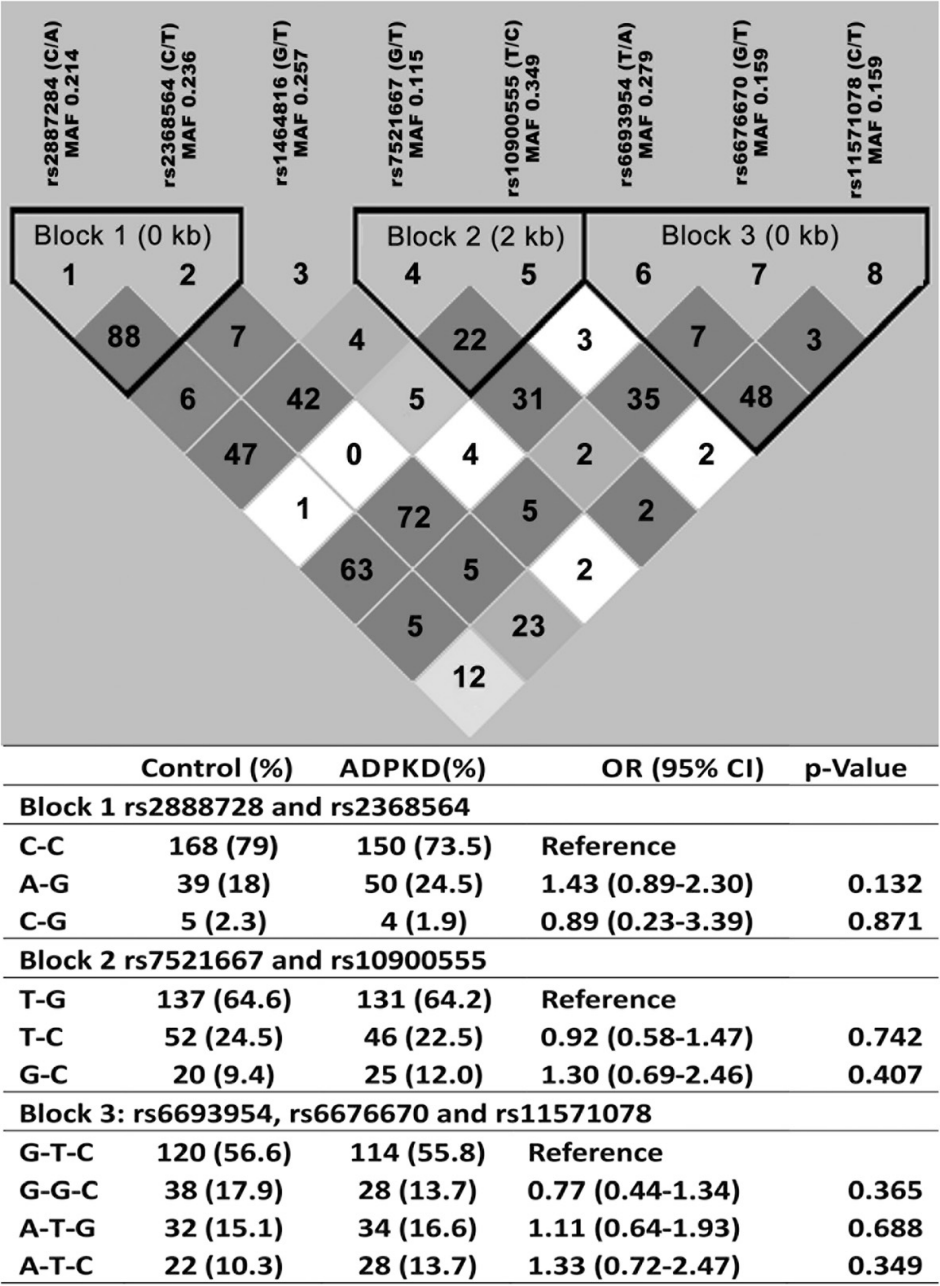
Pairwise linkage disequilibrium between the tag-SNPs of the *REN* gene. Pairwise LD measures (D and r^2^) were shown by the LD map. Square background color represents the D’/LOD and the values in cells are r^2^ values (multiplied by 100). The *REN* gene haplotypes distribution in control and ADPKD patients was shown in the bottom

The mean age of the control group was 53.27 ± 12.43 years and the ADPKD group was 46.89 ± 11.38 years. All tag-SNPs of the *REN* gene are polymorphic in both ADPKD and control groups and their distribution is documented in Table 1. Except for rs10900555, all tag-SNPs followed the Hardy–Weinberg Equilibrium in both control and ADPKD groups. The Cochran*-*Armitage trend test revealed that the distribution of the *REN* genotypes was not significantly different between control and ADPKD groups (Table 1). Results of pair-wise linkage disequilibrium (LD) analysis with these 8 SNPs are shown in Fig. 1. We observed three small haplotype blocks; first composed of rs2887284 and rs2368564; second of rs7521667 and rs10900555 and third block of rs6693954, rs6676670 and rs11571078. However, the SNP rs1464816 remained outside the haplotype blocks. Haplotype frequencies from each haplotype block were not significantly different between ADPKD and control groups (Fig. 1).

**Table 1:** Genotype distribution of REN gene tag-SNPs between control and ADPKD patients

Gene	Genotype	Control n (%)	ADPKD n (%)	OR (95 % CI)	*p*-Value (df-2)
rs2887284	CC	70 (66)	58 (56.8)	Reference	
	CA	33 (31.1)	38 (37.2)	1.39 (0.78–2.49)	
	AA	3 (2.83)	6 (5.8)	2.41 (0.58–10.08)	0.301
	MAF	18.4	24.5		
	HWE-p	0.703	0.945		
rs2368564	CC	65 (61.3)	54 (52.9)	Reference	
	TC	38 (35.8)	42 (41.1)	1.33 (0.75–2.35)	
	TT	3 (2.83)	6 (5.8)	2.41 (0.58–10.08)	0.343
	MAF	20.75	26.47		
	HWE-p	0.355	0.559		
rs1464816	GG	58 (54.7)	56 (54.9)	Reference	
	TG	40 (37.7)	41 (40.2)	1.06 (0.60–1.88)	
	TT	8 (7.5)	5 (4.9)	0.65 (0.20–2.10)	0.718
	MAF	26.4	25		
	HWE-p	0.762	0.467		
rs7521667	GG	85 (80.2)	76 (74.5)	Reference	
	TG	20 (18.8)	26 (25.5)	1.45 (0.75–2.81)	
	TT	1 (0.94)	0	-	0.430
	MAF	10.38	12.75		
	HWE-p	0.882	0.14		
rs10900555	TT	52 (49.0)	43 (42.1)	Reference	
	TC	35 (33.0)	46 (45.1)	1.59 (0.88–2.89)	
	CC	19 (17.9)	13 (12.7)	0.83 (0.37–1.87)	0.836
	MAF	34.4	35.3		
	HWE-p	0.005	0.898		
rs6693954	TT	56 (52.8)	48 (47.0)	Reference	
	TA	46 (43.4)	46 (45.1)	1.17 (0.67–2.05)	
	AA	4 (3.7)	8 (7.8)	2.33 (0.66–8.23)	0.283
	MAF	25.47	30.3		
	HWE-p	0.14	0.505		
rs6676670	GG	73 (68.8)	77 (75.5)	Reference	
	TG	28 (26.4)	22 (21.6)	0.75 (0.39–1.42)	
	TT	5 (4.7)	3 (2.94)	0.57 (0.13–2.47)	0.171
	MAF	17.9	13.7		
	HWE-p	0.292	0.367		
rs11571078	CC	76 (71.7)	71 (69.6)	Reference	
	TC	28 (26.4)	28 (27.4)	1.07 (0.58–1.98)	
	TT	2 (1.89)	3 (2.94)	1.61 (0.26–9.89)	0.659
	MAF	15.09	16.67		
	HWE-p	0.753	0.905		

RR: Relative risk; CI: confidence interval; MAF: minor allele frequency; HWp: Hardy-Weinberg *p* value; * *p-values* for the *Cochran-Armitage trend test*

Among ADPKD patients, 53 % showed early CKD stage with 51.8 ± 4.8 years of age while 47 % showed advanced CKD stage with a mean age of 35.8 ± 6.6. Univariate analysis showed that the distribution of *REN* tag-SNPs is not significantly different between early and advanced CKD groups (Table 2). None of the *REN* gene polymorphisms exhibited a confounding effect on the relationship between CKD progression and hypertension (Table 3). In multivariate analysis, the SNP rs1464816 showed significant association with increased CKD risk (TG vs. GG: OR = 4.80; 95 % CI = 1.30–17.82; *p* = 0.019) (Table 4), when corrected for traditional risk factors viz. age, hypertension and family history of diabetes mellitus.

**Table 2:** Effects of REN gene polymorphisms on CKD stages in ADPKD

Gene	Genotype	CKD stages		*p*-value*
		Early stage (*n* = 53)	Advance stage (*n* = 49)	
rs2887284	CC	30 (56.6)	28 (57.1)	
	CA	18 (33.9)	20 (40.8)	
	AA	5 (9.4)	1 (2.0)	0.509
rs2368564	CC	28 (52.8)	26 (53.0)	
	TC	20 (37.7)	22 (44.9)	
	TT	5 (9.4)	1 (2.0)	0.525
rs1464816	GG	33 (62.2)	23 (46.9)	
	TG	18 (33.9)	23 (46.9)	
	TT	2 (3.7)	3 (6.1)	0.131
rs7521667	GG	41 (77.3)	35 (71.4)	
	TG	12 (22.6)	14 (28.5)	
	TT	0 (0)	0 (0)	0.492
rs10900555	TT	25 (47.1)	18 (36.7)	
	TC	23 (43.3)	23 (46.9)	
	CC	5 (9.4)	8 (16.3)	0.199
rs6693954	TT	25 (47.1)	23 (46.9)	
	TA	21 (39.6)	25 (51.0)	
	AA	7 (13.2)	1 (2.0)	0.380
rs6676670	GG	43 (81.1)	34 (69.3)	
	TG	10 (18.8)	12 (24.4)	
	TT	0 (0)	3 (6.1)	0.076
rs11571078	CC	34 (64.1)	37 (75.5)	
	TC	16 (30.1)	12 (24.5)	
	TT	3 (5.6)	0 (0)	0.105

HT: Hypertension; FH-DM: Family history of diabetes mellitus; OR: odds ratio; CI: confidence interval. * *p-values* for the *Cochran-Armitage trend test*

**Table 3:** Association between CKD stages and hypertension stratified by REN genotypes

Gene	Genotype	OR (95 % CI for HT)	*p*-Value*
rs2887284	CC	3.0 (0.29–30.69)	
	CA	5.73 (1.00–32.67)	
	AA	0.75 (0.43–1.32)	0.36
M-H Combined		3.42 (0.97–12.07)	
rs2368564	CC	1.92 (0.16–22.56)	
	TC	6.67 (1.21–36.74)	
	TT	0.75 (0.43–1.32)	0.286
M-H Combined		3.42 (0.97–12.07)	
rs1464816	GG	3.94 (0.76–20.30)	
	TG	3.0 (0.48–18.65)	
	TT		0.828
M-H Combined		3.52 (1.04–11.90)	
rs7521667	GG	2.83 (0.53–15.02)	
	TG	8.4 (1.27–55.39)	
	TT	3.66 (1.10–12.13)	0.394
M-H Combined		4.47 (1.28–15.58)	
rs10900555	TT	1.09 (0.16–7.31)	
	TC	11.73 (1.33–103.79)	
	CC	4.67 (0.30–73.38)	0.227
M-H Combined		3.80 (1.16–12.48)	
rs6693954	TT	1.91 (0.16–22.63)	
	TA	4.60 (0.82–22.88)	
	AA	0.83 (0.58–1.19)	0.502
M-H Combined		2.84 (0.77–10.51)	
rs6676670	GG	3.55 (0.90–13.96)	
	TG	2.75 (0.21–35.84)	
	TT		0.863
M-H Combined		3.37 (1.01–11.26)	
rs11571078	CC	3.75 (0.70–20.03)	
	TC	2.27 (0.36–14.45)	
	TT		0.594
M-H Combined		3.01 (0.88–10.37)	

HT: Hypertension; M-H: *Mantel-Haenszel*

*Homogeneity test *p* value

**Table 4:** Adjusted effects of risk factors on CKD stages

Factors	OR (95 % CI)^a^	*p* value*
HT: Yes vs No	6.63 (1.38,31.81)	0.018
SEX: M vs F	0.45 (0.16,1.24)	0.123
Age: (40,60 year) vs, ≤40 year	6.71 (1.99,22.62)	0.002
Age: (60,90 year) vs, ≤40 year	15.3 (2.44,96.06)	0.004
FH-DM: Yes vs No	7.67 (2.54,23.14)	0.001
rs2887284:CA vs CC	2.41 (0.12–47.68)	0.563
rs2887284:AA vs CC	-	-
rs2368564: TC vs CC	0.68 (0.00–62.02)	0.87
rs2368564: TT vs CC	-	-
rs1464816: TG vs GG	4.80 (1.30–17.82)	0.019
rs1464816: TT vs GG	3.52 (0.25–48.93)	0.347
rs7521667: TG vs GG	5.00 (0.02–1004.64)	0.551
rs7521667: TT vs GG	-	-
rs10900555: TC vs TT	0.52 (0.73–3.80)	0.525
rs10900555: CC vs TT	0.13 (0.00–9.21)	0.352
rs6693954: TA vs TT	2.58 (0.62–106.83)	0.618
rs6693954: AA vs TT	-	-
rs6676670: TG vs GG	2.87 (0.38–21.60)	0.305
rs6676670: TT vs GG	-	-
rs11571078: TC vs CC	0.60 (0.00–51.79)	0.822
rs11571078: TT vs CC	-	-

^a^Adjusted for age, sex, hypertension (HT) and family history of diabetes (FH-DM)

OR: odds ratio; CI: confidence interval; **Wald test *p* value

## Discussion

Analysis of eight tag-SNPs within the *REN* gene did not show any significant association with ADPKD. Linkage disequilibrium analysis yielded three haplotype blocks and the haplotypes of the respective blocks are not statistically different between ADPKD and controls. However, the rs1464816 TG genotype showed a significant association with increased CKD risk in ADPKD in multivariate analysis. Earlier studies observed that the level of inactive renin found in normal plasma was significantly higher in uncomplicated diabetes mellitus and greatly increased in diabetic nephropathy [20, 21]. Further, the plasma of individuals with diabetic nephropathy showed increased levels of acid activated renin [22]. Furthermore, mice treated with direct renin inhibitor showed adipocyte differentiation and improved insulin sensitivity [23]. Plasma renin levels were decreased in the elderly regardless of the presence or absence of an inverse relationship with blood pressure [24]. In healthy subjects, beginning at puberty mean plasma renin activity and its levels decline with wide variations in individual values, and reach their lowest levels during the sixth decade of life [25].

Studies using animal models have clearly demonstrated the involvement of the renin gene in the development of hypertension [26, 27]. The hypertensive patients with polycystic kidney disease showed significantly higher plasma renin activity than patients with only essential hypertension [28]. Several polymorphisms within the renin gene or its flanking sequences that were studied for hypertension yielded inconsistent results [29–33]. The rs2368564 of the *REN* gene failed to show a significant association with hypertension in a Japanese population [13]. In contrast to this, the rs6693954 polymorphism showed higher plasma renin activity levels and was found to be associated with hypertension in the HyperPath cohort of Caucasian subjects [34].

Using renin antiserum and an immunoperoxidase method in nephrectomy and autopsy specimens of adult polycystic kidneys, the distribution of renin-containing cells was identified in residual normal kidneys, scarred renal parenchyma and areas of fibrous tissue [35]. As the juxtaglomerular apparatus is the main source of renin, abnormal distribution of renin-containing cells were identified in the juxtaglomerular apparatuses (JGAs) of nephrectomy and autopsy specimens*.* Further*,* hyperplasia of these cells in JGAs of untreated autopsy cases was documented [35]. Furthermore, synthesis of renin by tubulocystic epithelia was confirmed by different techniques [36]. ADPKD cyst-derived cells in culture revealed that the renin is expressed primarily in cysts of distal tubule origin and in cyst-derived cells with distal tubule characteristics [10]. In addition, radiolabelling of renin and mRNA for renin has been detected in cyst wall epithelia and cyst fluids [37]. Higher levels of plasma renin activity are associated with greater rates of CKD in hypertensive patients of an ethnically diverse population in southern California [38]. However, no study has been conducted to ensure the association of these polymorphisms with hypertension in ADPKD.

## Conclusion

In summary, our case–control study provides evidence that the polymorphism rs1464816 in *REN* gene is associated with CKD progression in ADPKD. The potential of the present study is limited, as we have not analyzed variations in *PKD1* and *PKD2* for ADPKD subjects although they were recruited based on clinical criteria. Therefore, further functional validation of these observational findings needs to be conducted. In addition, the nested study strategy adopted in this study may introduce selection bias and the small sample size used in this study is another limiting factor of statistical power. Lastly, the plasma renin activity levels were not determined and correlated with the progression of CKD as well as *REN* variants.

## Additional file

Additional file 1: Table S1. Primers used for genotyping *REN* tag-SNPs. (DOCX 13 kb)
