# Supplementary material for: Renin gene rs1464816 polymorphism contributes to chronic kidney disease progression in ADPKD
Source: J Biomed Sci. 2016 Jan 11;23:1. doi: 10.1186/s12929-015-0217-0 (PMC4710007; doi:10.1186/s12929-015-0217-0)
Supplement: Additional file 1: Table S1. — Primers used for genotyping REN tag-SNPs. (DOCX 13 kb) [file 12929_2015_217_MOESM1_ESM.docx]

**Table S1: Primers used for genotyping *REN* tag-SNPs.**

| Polymorphism | Primers^a^ | Sequence^b^ |
| --- | --- | --- |
| rs2887284 | Allele 1 (FAM) | 5'-CCATTTTCTCATCAAATACTCCCAAT-3' |
|  | Allele 2 (HEX) | 5'-CCATTTTCTCATCAAATACTCCCAAG-3' |
|  | Common reverse | 5'-AGAGGGTAAGAATATAGGAGCATAGGATT-3' |
| rs2368564 | Allele 1 (FAM) | 5'-AGAGTGACAGATTACAATGCACAGG-3' |
|  | Allele 2 (HEX) | 5'-AAAGAGTGACAGATTACAATGCACAGA-3' |
|  | Common reverse | 5'-GTGACATGCACAGCCAAGTTTGAGAA-3' |
| rs1464816 | Allele 1 (FAM) | 5'-GTGCGTGCAGGGTTGAGGC-3' |
|  | Allele 2 (HEX) | 5'-GTGTGCGTGCAGGGTTGAGGA-3' |
|  | Common reverse | 5'-GGGTACAGAAATCGGGGTAAGAGTA-3' |
| rs7521667 | Allele 1 (FAM) | 5'-CAAACCCATCCCAGGCACTC-3' |
|  | Allele 2 (HEX) | 5'-CTCAAACCCATCCCAGGCACTA-3' |
|  | Common reverse | 5'-GTAAGAGGTTGTTGATTAAGACTAGGGAT-3' |
| rs10900555 | Allele 1 (FAM) | 5'-AAGAAAGTAGAAGAAAGAGGGAATCTC-3' |
|  | Allele 2 (HEX) | 5'-GAAGAAAGTAGAAGAAAGAGGGAATCTT-3' |
|  | Common reverse | 5'-AAGTTGACTCTGCAATAGACTTTCCCATA-3' |
| rs6693954 | Allele 1 (FAM) | 5'-CAAAGAGGTTGCATTTCCCACTGA-3' |
|  | Allele 2 (HEX) | 5'-CAAAGAGGTTGCATTTCCCACTGT-3' |
|  | Common reverse | 5'-GTCCAAAGCATAGGCTGTTGTGAGTA-3' |
| rs6676670 | Allele 1 (FAM) | 5'-GAAAAACAGAGCTGAGGCCCAC-3' |
|  | Allele 2 (HEX) | 5'-GGAAAAACAGAGCTGAGGCCCAA-3' |
|  | Common reverse | 5'-CACCTCCAGGAAGCTTACCCCAA-3' |
| rs11571078 | Allele 1 (FAM) | 5'-GCATGATGCTCTGCACATAGCG-3' |
|  | Allele 2 (HEX) | 5'-CGCATGATGCTCTGCACATAGCA-3' |
|  | Common reverse | 5'-GGGGAATCCAGCTTTTGAAACAGTATTAT-3' |

^a^Primers corresponding to different alleles were labelled with FAM and HEX fluorescent dyes (KBiosciences).

^b^Polymorphic bases are underlined.
